# In the Shadow of Medicine: The Glaring Absence of Occurrence Records of Human-Hosted Biodiversity

**DOI:** 10.2196/60140

**Published:** 2024-12-09

**Authors:** Rémy Poncet, Olivier Gargominy

**Affiliations:** 1 Biodiversity & Geodiversity Knowledge Department, PatriNat (OFB, MNHN), 36 rue Geoffroy-Saint-HilaireParis, 75005, France, 33 0171214635

**Keywords:** human microbiome, bacterial occurrence data, public health, one health, biodiversity data gap, medical data integration, medical data, microbiome, bacterial, bacteria, biodiversity, disease prevention, pathogens, user-friendly, bacterial pathogens

## Abstract

Microbial diversity is vast, with bacteria playing a crucial role in human health. However, occurrence records (location, date, observer, and host interaction of human-associated bacteria) remain scarce. This lack of information hinders our understanding of human-microbe relationships and disease prevention. In this study, we show that existing solutions such as France’s Système d’Information sur le Patrimoine Naturel framework, can be used to efficiently collect and manage occurrence data on human-associated bacteria. This user-friendly system allows medical personnel to easily share and access data on bacterial pathogens. By adopting similar national infrastructures and treating human-associated bacteria as biodiversity data, we can significantly improve public health management and research, and our understanding of the One Health concept, which emphasizes the interconnectedness of human, animal, and environmental health.

## Introduction

Recent estimates place global diversity between 563 and 2206 million species, with bacteria being the most diverse, comprising between 364 and 1950 million species [1]. However, there is no synthetic data on the types of environments or (micro)habitats preferred by bacteria on a global scale. To gain an overview of the main environments colonized by microbial species (including bacterial, archaeal, and eukaryotic microbial species), researchers have used the three-level environment classification system associated with 23,828 samples collected during the Earth Microbiome Project as a proxy [2]. These data revealed that 52.32% of microbes are free-living (11.01% in saline environments and 88.99% in nonsaline environments), and the remaining 47.68% are host-dependent species (20.16% of plants and 79.84% of animals). Among the animal-derived samples, 49.88% originated from the gut, 32.64% from the body surface, 13.86% from secretions, and 3.62% from other parts of the body. Available data from other sources, such as MGnify [3], focus disproportionately on specific biomes for host-dependent species (as of March 9, 2024): humans (n=249,568), other mammals (n=54,432), plants (n=46,148), birds (n=8524), and insects (n=2921). However, von Meijenfeldt et al [4] used a balanced subsample of these data to show that biome annotations correlated relatively well with taxonomic profiles (at the order level), associated with sequence data, suggesting a possible biome-phylogeny relationship. Evidence of microbial niche differentiation further supports this notion [5].

The microbial diversity associated with the human species (*Homo sapiens*, Linnaeus 1758) is the subject of large-scale studies such as the Human Microbiome Project [6]. However, the data generated (mostly sequences) often lack taxonomic assignments or precise taxonomic assignments (eg, at the species level) that would enable specific microbe-host relationships (microbe species)–*(Homo sapiens*) to be robustly documented. In contrast, human pathogenic bacteria are better characterized taxonomically and assigned data at a detailed level, allowing these relationships to be established. For example, Bartlett et al [7] listed 1513 bacterial pathogens known to infect humans based on the literature available before 2021. This list includes *Chlamydia trachomatis* (Busacca 1935) Rake 1957; *Neisseria gonorrhoeae* (Zopf 1885) Trevisan 1885; and *Treponema pallidum* (Schaudinn and Hoffmann 1905) Schaudinn 1905, the three bacteria responsible for sexually transmitted infections—chlamydia, gonorrhea, and syphilis, respectively. In 2020, an estimated 128.5 million people were infected with chlamydia [8], 82.4 million with gonorrhea [9], and 7.1 million with syphilis [10]. In a report dated March 7, 2024, the European Centre for Disease Prevention and Control (ECDC) reported a significant increase in the number of sexually transmitted infection cases in 2022 compared to the previous year, with the number of gonorrhea cases increasing by 48% (70,881 confirmed cases) [11], syphilis cases by 34% (35,391 confirmed cases) [12], and chlamydia cases by 16% (216,508 confirmed cases) [13]. Despite these trends, we show that comprehensive occurrence data, including species, location, date, observer, and host-human interaction, available to the scientific, medical, and public health communities worldwide, is limited.

## Chlamydia, Gonorrhea, and Syphilis as Textbook Study Cases

**Figure 1.**
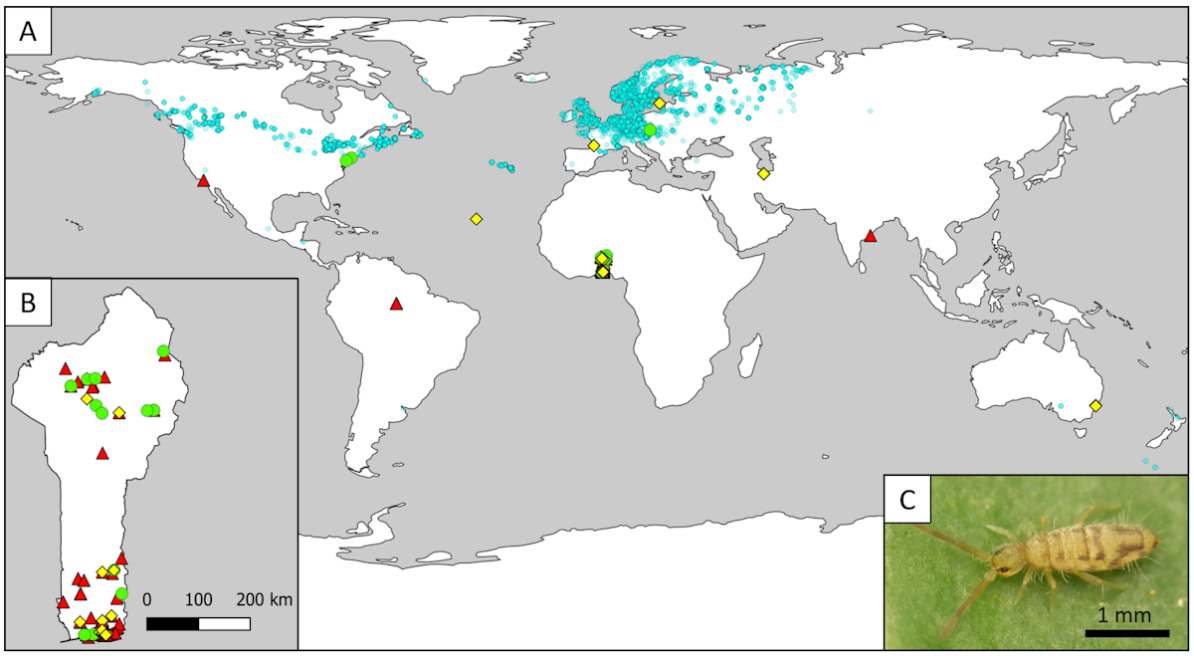
Available georeferenced records of three bacteria causing frequent sexually transmitted infections. (A) global distribution; (B) focus on Benin (which is the only country where occurrence records have been at least partially systematically collected between 2007 and 2020); (C) the habitus of the springtail *Entomobrya nivalis* (photo credit Alexis Orion) [19], which is published under Creative Commons Attribution 4.0 International License [20]. *Chlamydia trachomatis* is marked with yellow diamonds, *Neisseria gonorrhoeae* with red triangles, and *Treponema pallidum* with green circles. *Entomobrya nivalis* is depicted with small transparent turquoise circles. Note, the display of occurrence records for this springtail species is intended to highlight the lack of records concerning the three bacteria, despite their frequent occurrence globally.

The high prevalence of these three sexually transmitted infections at both the global [8-10] and European levels [11-13] serves as a textbook case for showing the degree of centralization and availability of occurrence records of human-associated bacterial species. Therefore, we searched for occurrence records of these three infectious bacterial species in the Global Biodiversity Information Facility (GBIF), and the results were surprisingly low: 28 georeferenced records for *Chlamydia trachomatis* [14], 205 for *Neisseria gonorrhoeae* [15], and 17 for *Treponema pallidum* [16]. Figure 1 shows the distribution of georeferenced records available in GBIF for these bacteria, compared with the springtail species *Entomobrya nivalis* (Linnaeus 1758) [17], which belongs to a taxonomic group with limited studies worldwide and benefits from a relatively small scientific community [18].

## The Ecosystem for Data Collection Already Exists

The transmission and centralization of occurrence records for any species (or at any other taxonomic level) requires at least three elements. The first concerns nomenclature, that is, the ability to properly write taxon names, to unambiguously designate the taxonomic concept under consideration at the time of taxonomic assignment of a specimen (regardless of the method used for specimen taxonomical assignation). Bacterial nomenclature is governed by the International Code of Nomenclature of Prokaryotes [21] and SeqCode [22], depending on the taxonomic description protocol employed. In practical terms, although taxon names can be found in the literature in which they were originally described, it is also possible to find their name (taxon and authority) in various databases such as the List of Prokaryotic names with Standing in Nomenclature [23], the Taxonomy Database of the National Center for Biotechnology Information [24], or the Catalogue of Life [25]. Second is the existence of data standards that guarantee formatting and enable interoperability between information systems. The Darwin Core standard [26], designed to provide a stable international standard reference for sharing information on biological diversity, can be used to record and disseminate data on the occurrence of human pathogenic bacteria. In particular, the standard enables the management of associated occurrences (one occurrence of pathogenic bacteria and one occurrence of host species) with the term “dwc:associatedOccurrences” and to qualify this association (eg, parasite of, host of) with the term “dwc:relationshipOfResource” (see ENETWILD consortium [27] and Astorga et al [28] for more details). Third is the access to software or applications for banking and managing species records (based on or compatible with GBIF-accepted data standards). This dimension is undoubtedly the most ambiguous one, since the availability of accessible services can vary depending on factors such as country, taxonomic group in question, stakeholders involved, etc. When such infrastructures do not exist, tools such as Symbiota [29], PlutoF [30], or EarthCape [31] can compensate for this gap. Additionally, optional elements can be added to these three dimensions to improve data reuse, in accordance with the FAIR (Findability, Accessibility, Interoperability, and Reusability) principles [32]. For example, data dictionaries can be designed using internationally recognized resources, such as the Earth Microbiome Project Ontology [33] or the European Molecular Biology Laboratory–European Bioinformatics Institute Ontology [34].

## An Example of the French Turnkey Ecosystem

**Figure 2.**
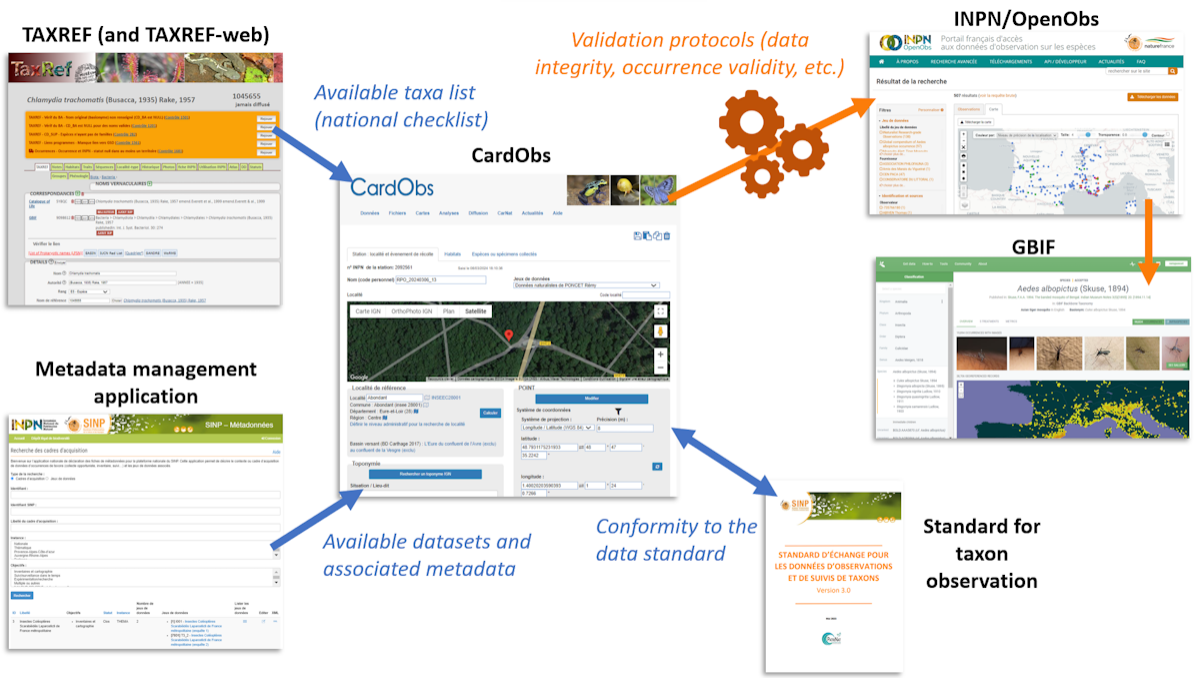
Overview of the services available in France for banking and making medical data available to understand human-related biodiversity. TAXREF feeds into CardObs with the official list of taxa and associated reference information. CardObs is connected to the metadata application, making it easy to select the dataset in which a record is entered. The CardObs information system complies with the official data model for taxon observations (GBIF-compatible). Once entered, the data undergo conformity (integrity) and qualification tests (level of validity of the data; procedure variable according to the reference systems available), and if they are compliant, they are uploaded to the INPN and then to the GBIF. GBIF: Global Biodiversity Information Facility; INPN: Inventaire National du Patrimoine Naturel.

Despite the limited number of occurrence records on human infectious bacteria in France, the solutions provided by the *Office français de la Biodiversité* and the *Muséum National d’Histoire Naturelle* offer a streamlined approach to manage all stages in the data cycle. This is achieved through the Système d’Information sur le Patrimoine Naturel (SINP) framework, which spans data acquisition to distribution via the GBIF. Figure 2 shows the main components of the SINP involved in this process:

- TAXREF, the national taxonomic repository [35] forms the backbone of the system, provided via the TAXREF-web application [36]. Its functionalities include (1) a taxonomic management application that supports taxon management, links with external Global Species Databases, and integration with Docs-Web [37], a bibliographic reference management system; (2) a taxon status management application that tracks official presence status across territories (eg, present, absent, extinct), and conservation and regulatory statuses developed by the International Union for Conservation of Nature Red List of Threatened Species; (3) a taxon trait management application for categorizing free-living species, host-dependent species, etc; (4) a taxon interaction management application that documents relationships (eg, *Chlamydia trachomatis* is an endoparasite of *Homo sapiens*); and (5) a habitat association management application related to the natural and seminatural habitats, based on the HABREF national repository [38].
- CardObs application [39,40] is designed to capture and manage taxon occurrence data. It is linked to the Metadata management application [41], which can be used to create datasets and specify parameters such as the data acquisition protocols. CardObs facilitates the creation of stations and the recording of one or more taxon occurrences for each of them. It is possible to establish relationships between taxa (eg, *Chlamydia trachomatis* was collected from *Homo sapiens*) and to link a record to a voucher or a sequence accession ID in repositories such as GenBank [42] or Barcode Of Life Data System [43]. Once entered, the data comply with the exchange standards for taxon observation and monitoring data [44] and undergo control procedures [45-47] before being entered into the Inventaire National du Patrimoine Naturel [48] (data accessible on the OpenObs platform [49]) and then into the GBIF.

In short, medical or laboratory staff identifying the presence of human parasitic bacteria in a sample must log on to CardObs (5 seconds), select the proper dataset (15 seconds), and enter the following minimum information: station (eg, patient’s city of residence or location of contamination, 15 seconds with autocomplete), date of record (15 seconds), data collector (10 seconds); taxon (eg, bacterial species, 20 seconds with spelling check); related species (eg, *Homo sapiens*, between 0 to 15 seconds depending on the settings for the specific field, which may have only one entry by default: *Homo sapiens*); and the type of link between the two species (eg, bacteria is a pathogen associated with *Homo sapiens*, 0 to 15 seconds, depending on the settings for the specific field, which may have only one entry by default: pathogen). This entry, which takes between 1 minute and 30 seconds to 2 minutes (depending on the experience of the user and the setting of some fields), would help to fill the gap in occurrence data on bacteria linked to humans in a very simple and efficient way and would provide valuable data to support research and public health (alert, prevention, control, guiding public health policies, etc). As an example, if each hospital (n=2983) and each medical biology laboratory (n=426) in France dedicated 8 hours a year to this task, it could result in approximately 800,000 records per year (assuming 2 minutes to enter one occurrence data and sufficient records for 8 hours).

## Discussion

The lack of systematic banking and dissemination of data on human-hosted species (bacteria, parasites, etc) suggests three facts: (1) the lack of consideration of the human species as an ecosystem hosting biodiversity; (2) the failure to consider data generated in a medical context as data of interest regarding biodiversity, environment, and the One Health concept; and (3) the absence of a policy (or any other kind of incentive) for the systematic banking of these data (occurrence, species-species relationship), limiting their availability to the scientific community and society. Raising awareness about the benefits of banking such data to make them accessible and reusable (which is possible due to the existing solutions addressing the entire data life cycle) could support research and disease prevention in the field of public health and human-environment interactions. While we are not aware of any study that relies on such open-access big data–since they are currently rare or poorly identified as sources of interest, many scenarios could generate valuable data or would have benefited greatly from the prior banking of human pathogen occurrence data. For example, (1) documenting seasonal variations in human contamination by *Escherichia coli* (Migula 1895) Castellani and Chalmers 1919, originating from reindeer in regions with high tourist numbers [50]; (2) assessing the link between the risk of bacterial contamination from seafood, urbanization, and rainy weather events [51]; or (3) identifying the relationships between air pollution levels, seasonality, and transmission of antibiotic-resistant pathogens [52]. Moreover, ECDC reports on the three pathogenic bacteria in the study case indicate that countries such as France, Germany, Belgium, the Netherlands, Austria, and Italy were unable to systematically transfer summary data for the period 2018 to 2022 due to inadequate data collection systems. In addition, the lack of centralization of human infections of bacterial origin hinders the potential exploitation of big data for biodiversity studies. For example, the three bacteria used in this study case are known to interact with other species that coexist with the human species in many localities; for example, *Chlamydia trachomatis* with house mouse (*Mus musculus*, Linnaeus 1758), long-tailed macaque (*Macaca fascicularis*, Raffles 1821), and wild boar (*Sus scrofa*, Linnaeus 1758) [53], *Neisseria gonorrhoeae* with house mouse (*Mus musculus*) [54], and *Treponema pallidum* with several monkey species [55]. Host species of these pathogenic bacteria collectively have over 500,000 occurrence records in the GBIF [56]. Thus, in line with the implementation of the One Health concept [57-59] and in compliance with the provisions of the Aarhus Convention [60]–specifically, articles “4. Access to environmental information” and “5. Collection and dissemination of environmental information”–encouraging the banking and availability of biodiversity data generated in the medical sector would represent a significant advancement. This initiative would enhance public health management, epidemic prevention, research on topics such as pathogen transmission, antibiotic resistance, interplay between infection and the state of the environment, etc. As the technology and information systems already exist (or are easily adaptable) in the majority of countries contributing to GBIF [61], the lack of data banking for human-associated pathogenic bacteria is the result of a lack of leadership (scientific, technical, and political) on this subject and is detrimental for public health and research assessing the human-environment relationship. Biodiversity experts have succeeded in implementing such data collection and centralization on a global scale, accumulating close to 3 billion data records globally [62]—there is no reason why the medical and public health sectors cannot do the same.

## Abbreviations

ECDC: European Centre for Disease Prevention and Control
FAIR: Findability, Accessibility, Interoperability, and Reusability
GBIF: Global Biodiversity Information Facility
SINP: Système d'Information sur le Patrimoine Naturel (Natural Heritage Information System)
